# Supplementary figures and images for: Metabolic Modeling of Hermetia illucens Larvae Resource Allocation for High-Value Fatty Acid Production
Source: Metabolites. 2023 Jun 3;13(6):724. doi: 10.3390/metabo13060724 (PMC10304575; doi:10.3390/metabo13060724)

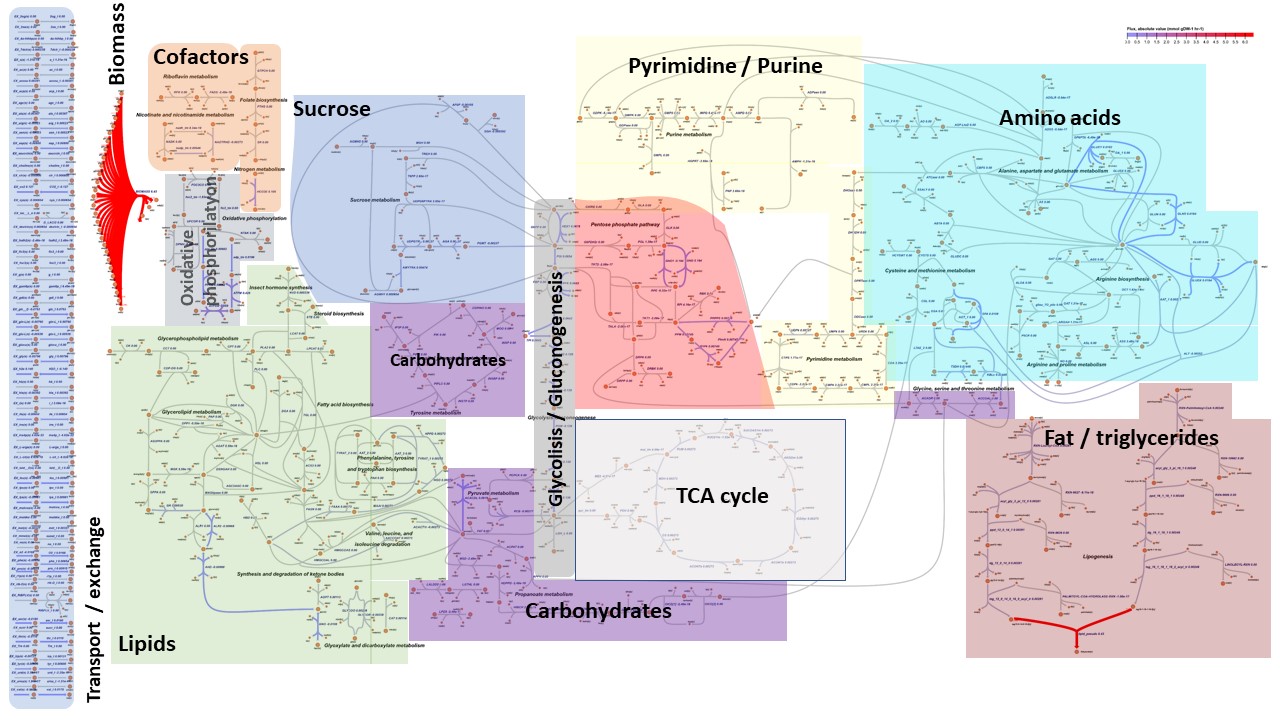

Supplement: Supplementary file 1 [file metabolites-13-00724-s001.zip › metabolites-2327137-supplementary/Hermetia_illucens_metabolic_model-main/7_Hermetia_network_Fig_5.jpg]
