# Supplementary material for: Metabolic Modeling of Hermetia illucens Larvae Resource Allocation for High-Value Fatty Acid Production
Source: Metabolites. 2023 Jun 3;13(6):724. doi: 10.3390/metabo13060724 (PMC10304575; doi:10.3390/metabo13060724)
Supplement: Supplementary file 1 [file metabolites-13-00724-s001.zip › metabolites-2327137-supplementary/Hermetia_illucens_metabolic_model-main/1_Supplemental_information..pdf.zip!/1_Supplemental_information..pdf]

1. File of Supplemental table of content.
2. *H. illucens* medium scale metabolic model in JSON file format (Compatible with CobraPy)
3. *H. illucens* medium scale metabolic model in MATLAB Mat file format (Compatible with Cobra Toolbox 3.0)
4. *H. illucens* medium scale metabolic model in XLSX file format (Compatible with Cobra Toolbox 3.0 with xlsx import support)
5. *H. illucens* medium scale metabolic model in SBML (XML) file format compatible with other metabolic modelling tools.
6. *H. illucens* medium scale metabolic model interactive biochemical pathways interactive map, which is compatible with IMFler interactive online modelling tool.
7. *H. illucens* medium scale metabolic model interactive biochemical pathways map in JPG file format
8. *H. illucens* Larva and diet composition experimental measurements
9. *H. illucens* Larva calculated biomass function measurements
10. *H. illucens* Chemcial analysis experimentals measurements for larva and diet composition (preliminary data)
